# Preservation of the Posterior Meningeal Artery During Excision of a Foramen Magnum Meningioma: A Case Report

**DOI:** 10.1002/ccr3.73573

**Published:** 2026-09-21

**Authors:** Eiji Ito, Yasuhiro Nakajima, Takashi Izumi, Takashi Tsujiuchi, Ayako Motomura, Ryuta Saito

**Affiliations:** ^1^ Skull Base Surgical Center Aichi Medical University Hospital Nagakute Aichi Japan; ^2^ Department of Neurosurgery Daido Hospital Nagoya Aichi Japan; ^3^ Department of Neurosurgery Nagoya University Graduate School of Medicine Nagoya Aichi Japan

**Keywords:** brainstem infarction, meningeal artery, meningioma, motor‐evoked potential, vertebral artery

## Abstract

A 56‐year‐old woman presented with a foramen magnum meningioma associated with headache and left hypoglossal nerve palsy. A vessel arising from the intradural vertebral artery was identified as the posterior meningeal artery based on an integrated assessment of its origin, course, and relationship to the tumor on three‐dimensional angiography and at surgery. During temporary occlusion, the left abductor pollicis brevis motor‐evoked potential (MEP) was present at 8 min and absent at 9 min. It was again detectable in the recording obtained 7 min after clip release, but subsequent responses were not consistently discernible. Stimulation intensity had been increased after signal loss, and dissection continued during occlusion, limiting attribution of the MEP changes to vessel occlusion or release. The vessel was preserved, and Simpson Grade IV resection was performed. Histomorphology supported meningothelial meningioma, CNS WHO Grade 1; the Ki‐67 labeling index was 1%. This case illustrates vessel‐preservation decisions informed by anatomical correlation and repeated neurophysiological assessment when vascular function is uncertain.

## Key Clinical Message

During foramen magnum meningioma resection, a meningeal feeder may warrant preservation when its functional significance is uncertain. In this case, a motor‐evoked potential response was absent at 9 min of temporary occlusion. Although stimulation changes and concurrent dissection limited causal interpretation, anatomical correlation and repeated intraoperative monitoring supported vessel preservation.

## 1 Introduction

The posterior meningeal artery (PMA) typically arises from the V3 segment of the vertebral artery (VA), near or just below the foramen magnum, and predominantly supplies the posterior fossa dura [1]. It may supply posterior fossa tumors or dural arteriovenous fistulas and can participate in unusual collateral pathways [2, 3, 4, 5, 6, 7]. Understanding its origin, course, and potential anastomoses is therefore important when embolization or surgical interruption is considered.

Anastomoses with other meningeal arteries may maintain dural supply after proximal PMA interruption [1]. Connections with the posterior inferior cerebellar artery (PICA) or lateral spinal artery, however, may create a risk of cerebellar or brainstem ischemia [2, 8, 9]. Foramen magnum meningiomas (FMMs) are surgically challenging because of their proximity to the brainstem, lower cranial nerves, and posterior circulation [10, 11]. This report describes an FMM in which loss of a motor‐evoked potential (MEP) response during temporary occlusion of the PMA prompted vessel preservation.

## 2 Case History/Examination

A 56‐year‐old woman with no remarkable medical history underwent evaluation for headaches and was found to have an intracranial neoplastic lesion. Clinical examination revealed left hypoglossal nerve palsy, but no other neurological deficits were noted.

**FIGURE 1:**
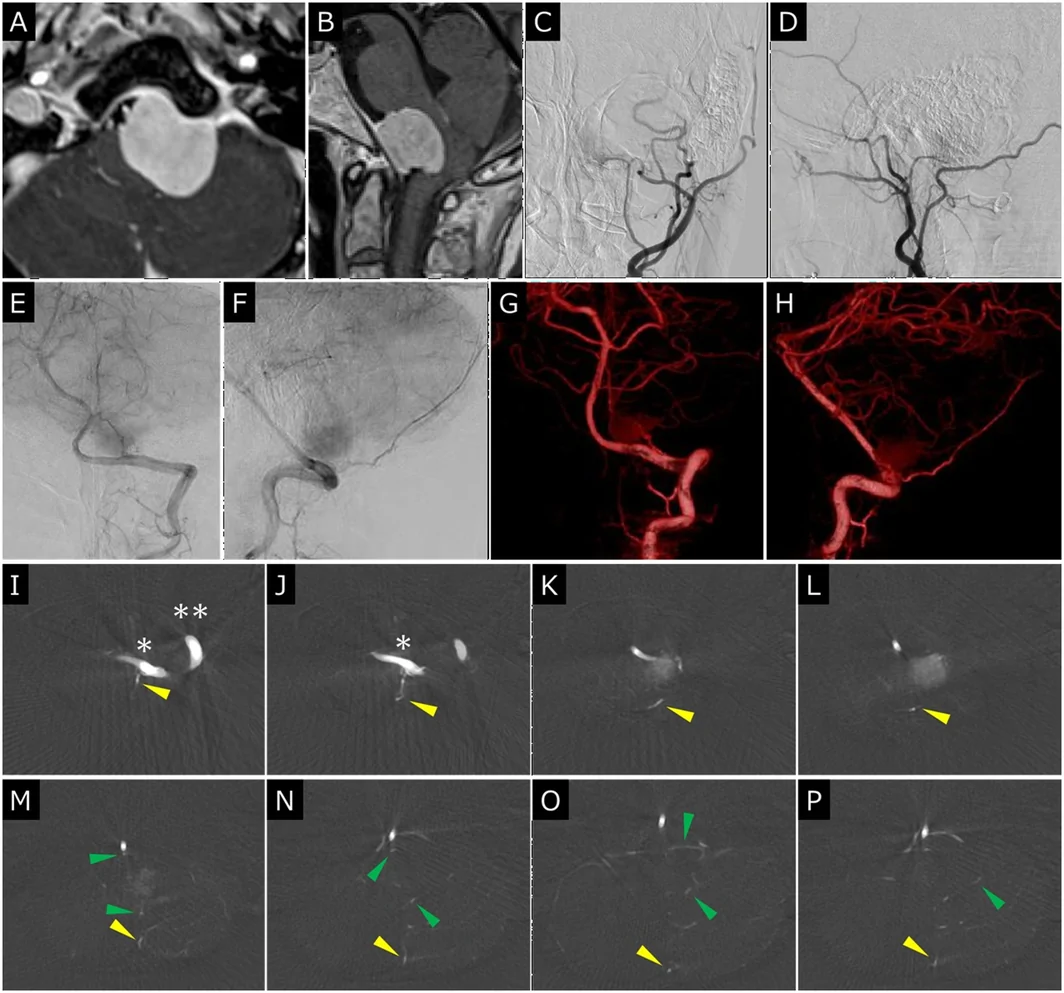
(A) Preoperative T1‐weighted MRI with gadolinium enhancement shows an extra‐axial mass. (B) The lesion is attached to the left lower clivus and compresses the medulla oblongata. (C, D) Left external carotid angiography (anteroposterior and lateral views) shows tumor staining from the ascending pharyngeal artery. (E, F) Left vertebral angiography (anteroposterior and lateral views) shows tumor staining from the PMA. (G, H) Three‐dimensional left vertebral angiography. (I–L) Thin‐slice reconstructions of three‐dimensional rotational digital subtraction angiography show the PMA arising from the V4 segment of the VA (I, yellow arrowhead), staining the tumor while coursing through the left medullary subarachnoid space (J), traversing toward the posterior fossa dura dorsal to the foramen magnum (K), and ascending near the internal occipital crest (L). (M–P) Sequential thin‐slice reconstructions show separate origins of the PMA and PICA from the VA. *: Intradural segment of the VA; **: Atlantic segment of the VA; MRI: Magnetic resonance imaging; PICA: Posterior inferior cerebellar artery; PMA: Posterior meningeal artery; VA: Vertebral artery; yellow arrowhead: PMA; green arrowhead: PICA.

Magnetic resonance imaging (MRI) revealed a gadolinium‐enhancing extra‐axial mass on T1‐weighted images (Figure 1A) that was hypointense on T2‐weighted images. The lesion was attached to the left lower clivus and compressed the medulla oblongata (Figure 1B). Three‐dimensional computed tomography angiography showed caudal displacement of the VA by the tumor. Cerebral angiography showed tumor staining from the ascending pharyngeal artery and a meningeal branch arising from the intradural VA, identified as the PMA (Figure 1C–H). Thin‐slice reconstructions of three‐dimensional rotational digital subtraction angiography showed that this branch originated from the V4 segment of the VA and prominently supplied the tumor (Figure 1I–L). Sequential reconstructions showed a separate PICA arising more cranially from the V4 segment (Figure 1M–P). These observations supported the distinction of the PMA from the separately visualized PICA.

## 3 Methods

Tumor removal through a transcondylar approach with preoperative embolization of feeding arteries was planned. Only the ascending pharyngeal artery was embolized because the PMA was expected to be accessible during craniotomy. Under total intravenous anesthesia, an endotracheal tube with electrodes was used. The patient was placed in the park‐bench position with head flexion, vertex‐down positioning, rotation, and head‐pin fixation. Transcranial motor‐evoked potentials (MEPs), somatosensory evoked potentials (SEPs), auditory brainstem responses (ABRs), and cranial nerve monitoring of the bilateral vagus, left facial, and left hypoglossal nerves were used. For MEP monitoring, stimulation was set at approximately 120% of the threshold at which upper‐extremity responses from the abductor pollicis brevis (APB) were reliably elicited, rather than at supramaximal intensity. At this control intensity (typically approximately 100 mA), upper‐extremity MEPs were consistently obtained, whereas lower‐extremity responses from the abductor hallucis (AH) were not consistently elicited.

Subsequently, a horseshoe‐shaped skin incision was made. The V3 segment of the VA with the vertebral venous plexus was exposed. C1 hemilaminectomy and partial condylectomy were performed; the occipital condyle was exposed, and approximately one‐third of the medial side was osteotomized to avoid damage to the articular surface of the condyle by epidural manipulation.

**FIGURE 2:**
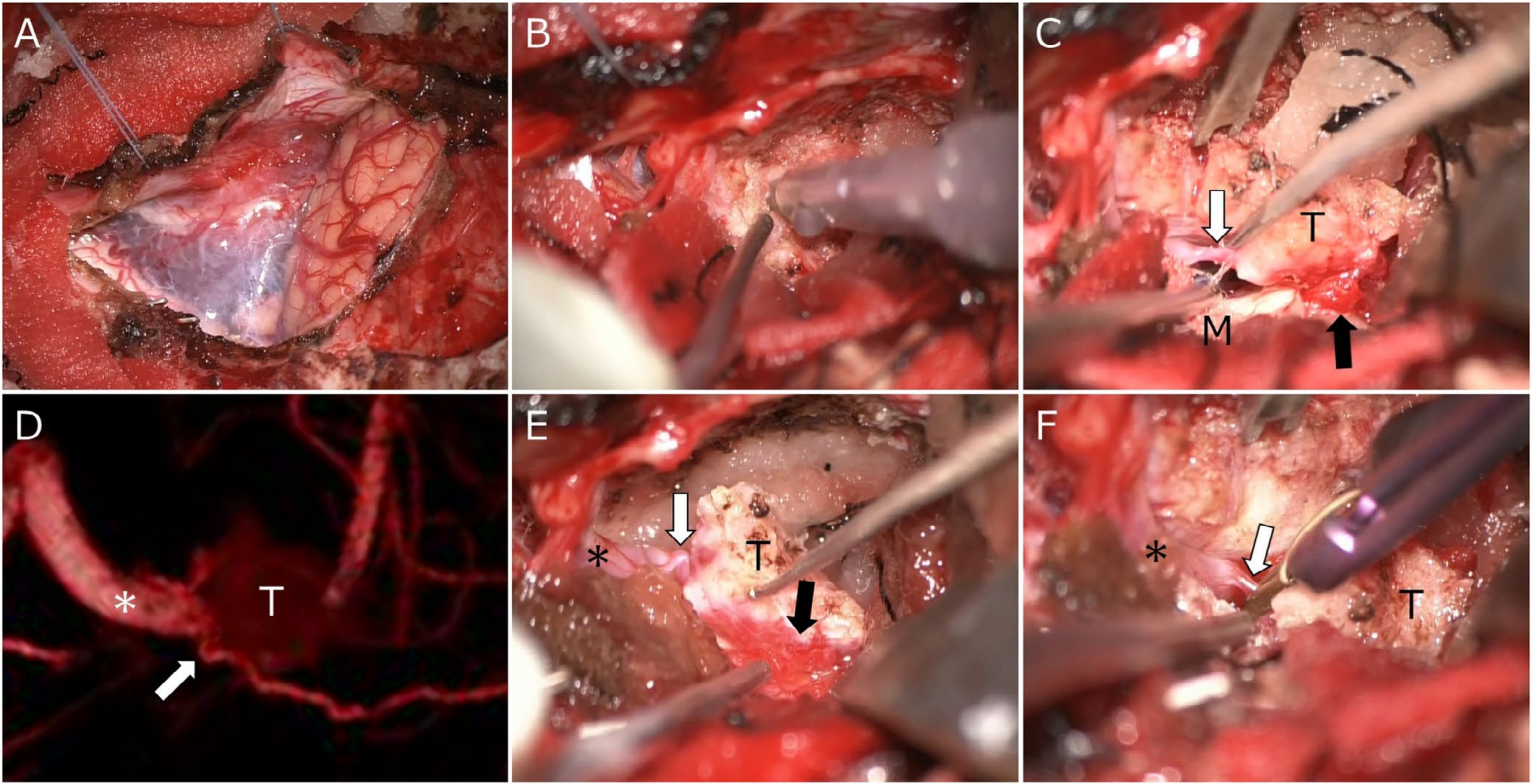
Correlation of angiographic and intraoperative anatomy. (A) After dural incision, the tumor is attached primarily to the jugular tubercle. (B) Tumor debulking with a Cavitron Ultrasonic Surgical Aspirator. (C) Intraoperative vessel arising from the intradural VA (white arrow). (D) Three‐dimensional angiography reconstructed from approximately the operative viewing angle shows the corresponding PMA (white arrow). The branch origin, course, and tumor relationship correspond between C and D. The separately arising PICA is shown in Figure 1M–P. This anatomical correspondence supports PMA identification but does not demonstrate neural perfusion. (E) The PMA (white arrow) appears to reach the tumor periphery and medullary surface. The black arrows in C and E indicate a suspected surface connection, not a proven pial anastomosis. (F) Temporary clip occlusion of the PMA (white arrow). *: VA; M: Medulla oblongata; PMA: Posterior meningeal artery; T: Tumor; VA: Vertebral artery.

After dural incision, the cerebellum was retracted cranially. The tumor was attached primarily to the jugular tubercle (Figure 2A), surrounded by an arachnoid membrane, and relatively well demarcated from the cerebellum. The accessory cranial nerves coursed dorsally without tumor adhesion. The tumor was internally debulked with a Cavitron Ultrasonic Surgical Aspirator and removed piece by piece (Figure 2B), after which residual tumor was dissected from the brainstem. The VA contacted the tumor caudally, and a vessel arising from the intradural VA was identified (Figure 2C). Three‐dimensional angiography was reconstructed from approximately the operative viewing angle (Figure 2D). The branch origin from the intradural VA, direction of travel, and relationship to the tumor matched between panels C and D, and the PICA had a separate origin. Based on an integrated assessment of these angiographic and intraoperative findings, we identified the vessel as the PMA. Selective PMA imaging and intraoperative angiography were not performed.

**FIGURE 3:**
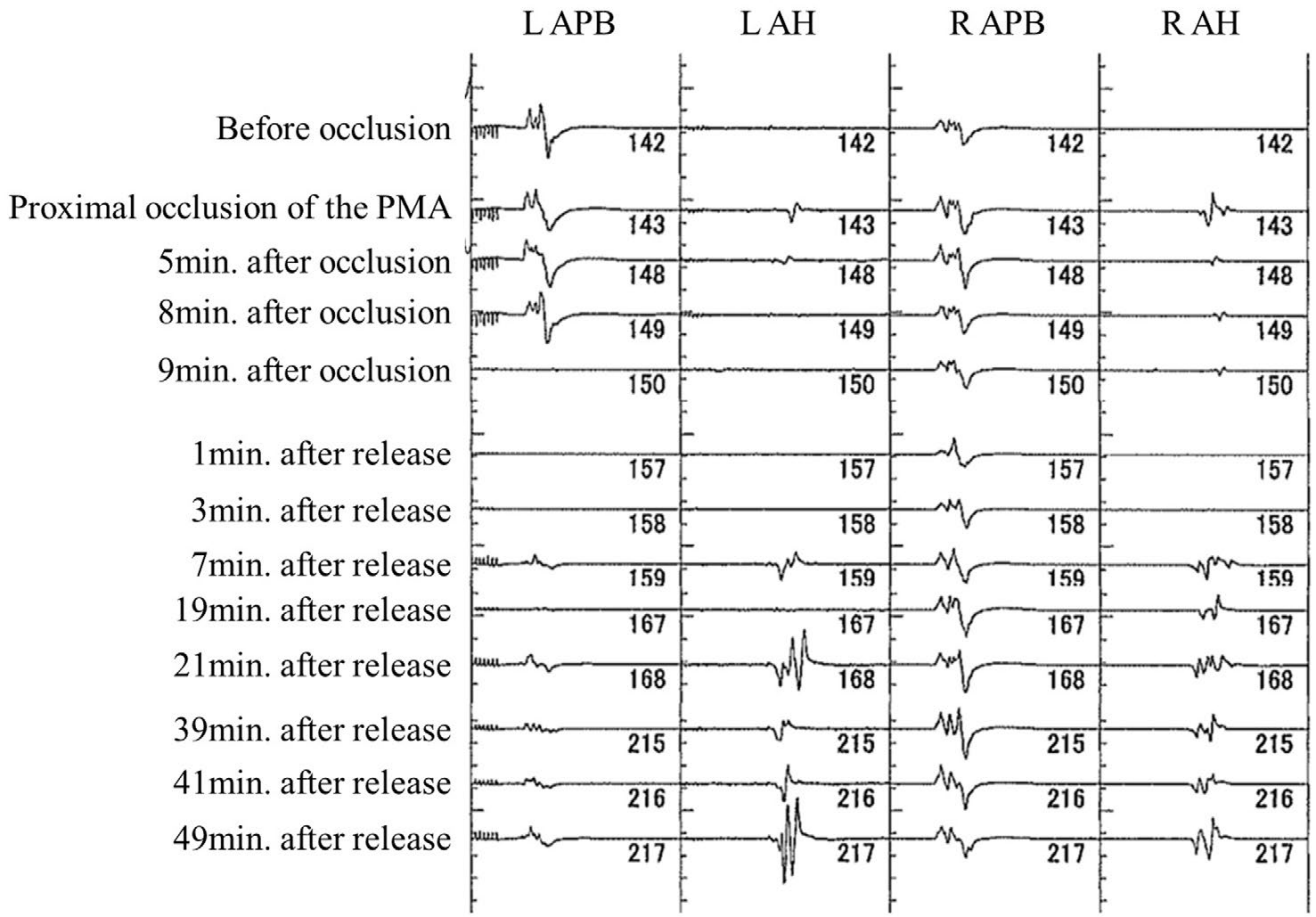
Motor‐evoked potential recordings during temporary PMA occlusion and after clip release. The left APB response is present at 8 min and absent at 9 min of occlusion. It is detectable at 7 min after release, but subsequent responses are not consistently discernible; the 19‐min trace appears nearly flat. Reappearance does not establish sustained or baseline recovery. The right APB response remains detectable, with variation across recordings. Bilateral AH responses are inconsistently elicited at the control intensity (approximately 120% of the upper‐extremity MEP threshold). Stimulation was increased after left APB loss, and AH responses subsequently became detectable. Exact stimulation settings for each trace could not be verified; amplitudes across the change are not directly comparable. AH: Abductor hallucis; APB: Abductor pollicis brevis; L: Left; MEP: Motor‐evoked potential; PMA: Posterior meningeal artery; R: Right.

The vessel appeared to be a tumor feeder and was initially considered for division. Careful inspection suggested that it reached both the tumor periphery and the medullary surface; temporary clip occlusion was therefore performed (Figure 2E,F). The surgeon continued dissecting other portions of the tumor while the clip remained in place. Our usual practice was to reassess MEP responses over approximately 5 min before considering vessel division; this was an operative practice rather than a validated safety threshold for the PMA. MEPs were repeatedly assessed during continued occlusion. In the displayed recordings, the left APB response was present at 8 min and absent at 9 min (Figure 3). The right APB response remained detectable, with variation in amplitude across subsequent recordings. Bilateral AH responses were inconsistently elicited at the control intensity and were not used as the primary endpoint. After loss of the left APB response, stimulation was increased above the control intensity; AH responses subsequently became detectable under the different stimulation conditions.

The left APB response was again detectable in the recording obtained 7 min after clip removal, and the right APB response remained present (Figure 3). The subsequent displayed left APB traces did not show a consistently discernible response; the trace at 19 min after release appeared nearly flat. Reappearance did not establish sustained recovery or restoration of the pre‐occlusion amplitude under identical stimulation conditions. The MEP warning and the vessel's relationship to the medulla supported preservation. Postoperative review of the angiographic reconstructions suggested that, after reaching the tumor, the PMA traversed the left medullary subarachnoid space toward the posterior fossa dura dorsal to the foramen magnum (Figure 1J–L).

## 4 Results and Outcome

**FIGURE 4:**
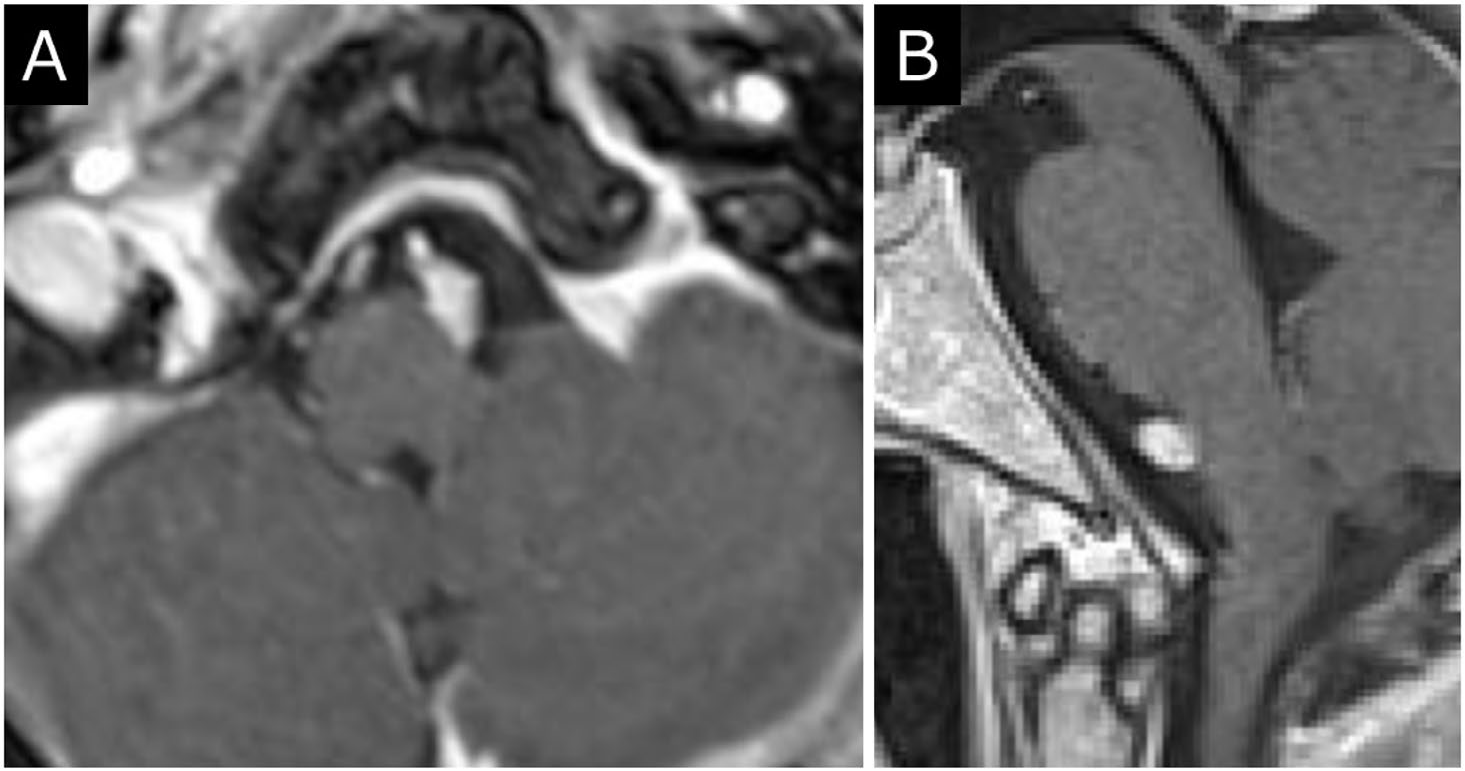
(A, B) Postoperative MRI shows residual tumor on the ventral surface of the medulla oblongata. MRI: Magnetic resonance imaging.

**FIGURE 5:**
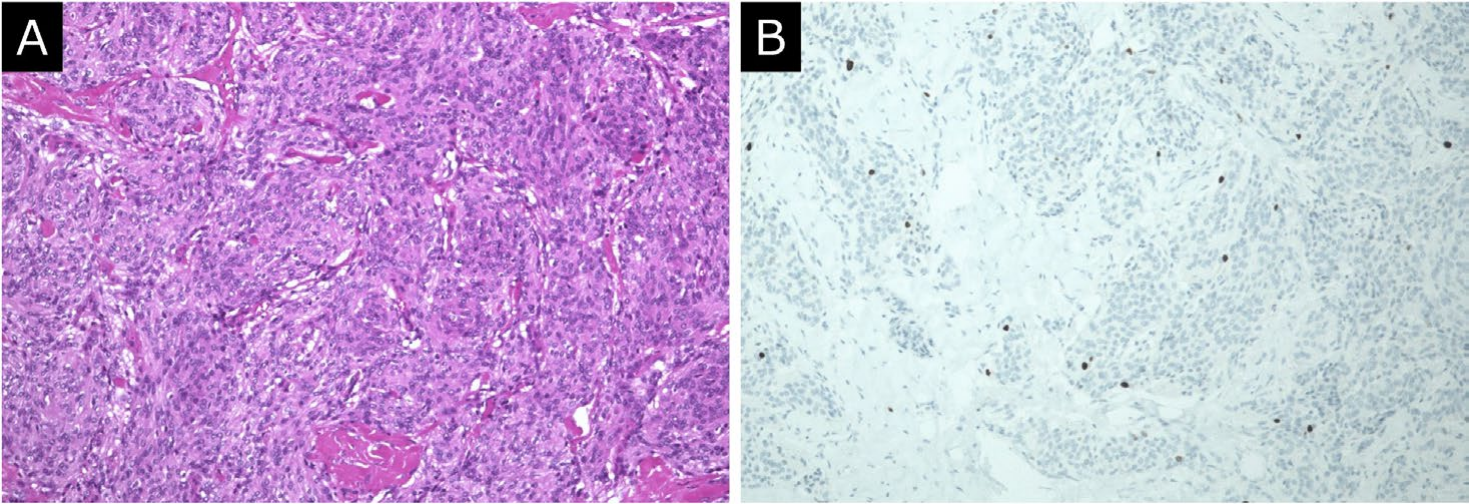
Histopathological findings. (A) H&E staining shows polygonal cells with eosinophilic cytoplasm and round‐to‐oval nuclei arranged in syncytial nests and meningothelial whorls within a lobulated tumor with fibrous stroma, supporting meningothelial meningioma. (B) Ki‐67 immunohistochemistry shows sparse nuclear labeling; the reported labeling index was 1%. H&E: Hematoxylin and eosin.

Postoperatively, the patient's headache resolved completely. The left hypoglossal nerve palsy persisted, but no new neurological deficit occurred. Simpson Grade IV resection was achieved: The dural attachment at the jugular tubercle was coagulated, whereas tumor on the ventral medullary surface was intentionally left to preserve the PMA and avoid brainstem injury. Postoperative MRI showed the residual tumor (Figure 4A,B). Histopathological examination of hematoxylin and eosin (H&E)‐stained sections showed a lobulated tumor with fibrous stroma. Polygonal cells with eosinophilic cytoplasm and round‐to‐oval nuclei were arranged in syncytial nests and meningothelial whorls (Figure 5A). Nuclear pleomorphism and mitotic figures were not identified in the examined sections. The syncytial architecture and meningothelial whorls favored meningioma over schwannoma and solitary fibrous tumor, supporting the morphological diagnosis of meningothelial meningioma, CNS WHO Grade 1. Ki‐67 immunohistochemistry showed a labeling index of 1%, indicating low proliferative activity (Figure 5B). MRI showed no tumor progression 2 years after surgery.

## 5 Discussion

The central issue in this case was whether the tumor‐feeding PMA could be safely divided. Its relationship to the medullary surface and the loss of the left APB MEP response during temporary occlusion supported vessel preservation, although concurrent dissection and changes in stimulation limited causal interpretation of the monitoring findings.

## 6 PMA Anatomy Supports Preservation When Function Is Uncertain

The concordant angiographic and operative anatomy (Figure 2C,D), together with the separately arising PICA (Figure 1M–P), supported PMA identification. In a series of 300 angiographic cases, PMA origin was classified as type A (from the distal PICA), type B (from the extracranial VA with entry through the foramen magnum), or type C (directly from the intracranial VA); type C accounted for 1% of cases [9]. The intradural VA origin and subarachnoid course in our patient were compatible with type C, although this classification remained provisional. Lateral medullary infarction has been reported during PMA embolization, with suspected occlusion of a medullary perforator during microcatheter/guidewire manipulation [8]. This report highlights the risk of injury to a medullary branch arising from the PMA. Reports of PMA‐pial collateral pathways [4, 5, 12] and PMA origin from the lateral medullary segment of the PICA [2] provide further anatomical context for this concern. In our patient, however, nonselective imaging and the vessel's surface relationship did not demonstrate a patent pial anastomosis or establish a neural perfusion territory. These anatomical concerns supported preservation when considered alongside the intraoperative monitoring warning.

## 7 Intraoperative Monitoring and Surgical Decision‐Making

The selection and interpretation of intraoperative monitoring are challenging near the foramen magnum. Injury to a medullary branch arising from the PMA may cause lateral medullary ischemia and Wallenberg syndrome [8], yet focal ischemia may escape standard MEP, SEP, or ABR monitoring. Experimental medullary trigeminal‐evoked potentials have been investigated as a complementary monitoring approach [13], but their application differs from routine MEP monitoring.

The left APB response was more affected than the right despite the left‐sided tumor. At the hypoglossal canal level, corticospinal fibers are generally predecussational [14], so a focal left‐sided lesion at that level would ordinarily affect right‐sided motor responses. Involvement of fibers at or caudal to the pyramidal decussation is a possible explanation for the left‐sided change. However, neither the affected tissue nor its relationship to the decussation was demonstrated in this patient. The laterality therefore remains unresolved.

MEP monitoring can detect impaired blood supply to motor pathways during middle cerebral artery aneurysm surgery [15], but evidence from that setting does not validate a 5‐min threshold for permanent interruption of the PMA. In this case, the clip remained in place while dissection continued elsewhere; the left APB response was present at 8 min and absent at 9 min. The timing was an incidental observation during continued occlusion, not a planned prolonged test. This case supports repeated assessment during ongoing occlusion but cannot define a safe observation period, establish tolerance of permanent division, or justify deliberately prolonging an occlusion test.

The MEP sequence provides a temporal association rather than proof of an ischemic mechanism. Dissection continued during occlusion, and stimulation was increased after APB loss. Exact per‐trace stimulation settings, equivalence of pre‐ and post‐release conditions, and contemporaneous hemodynamic, anesthetic, or operative changes could not be verified retrospectively. Signal reappearance therefore cannot be attributed to clip release alone or taken as confirmed restoration of baseline amplitude. These limitations also prevent the MEP findings from establishing that the PMA supplied the affected motor pathway.

## 8 Surgical Outcome

The transcondylar approach carries risks of lower cranial nerve dysfunction, VA injury, cerebrospinal fluid leakage, and craniocervical instability [11, 16]. The patient's pre‐existing left hypoglossal nerve palsy persisted postoperatively. Resection of approximately the medial one‐third of the occipital condyle provided adequate exposure while limiting the risk of instability.

Simpson Grade IV resection was selected because further removal from the ventral medullary surface would have risked injury to the PMA and brainstem. In a multi‐institutional series of 51 patients with FMMs, VA involvement was associated with postoperative complications [16]. This finding contextualizes vascular risk during FMM surgery but does not establish the functional significance of the PMA in this patient. MRI showed no progression at 2 years; continued surveillance is planned, with radiosurgery reserved for documented progression. This case illustrates a preservation decision under uncertainty and cannot establish a general occlusion protocol.

## Author Contributions

**Eiji Ito:** writing – original draft, writing – review and editing. **Yasuhiro Nakajima:** writing – original draft. **Takashi Izumi:** writing – original draft, writing – review and editing. **Takashi Tsujiuchi:** writing – original draft. **Ayako Motomura:** writing – original draft. **Ryuta Saito:** writing – review and editing.

## Funding

The authors have nothing to report.

## Ethics Statement

This study was performed in accordance with the ethical standards of the Japan Neurosurgical Society.

## Consent

Written informed consent was obtained from the patient for the publication of this case report and the accompanying data and images.

## Conflicts of Interest

The authors declare no conflicts of interest.

## Data Availability

The de‐identified clinical data and images supporting this report are available to the journal and its reviewers from the corresponding author upon reasonable request. They are not publicly available to protect patient privacy.
